# Nanostructured gold microelectrodes for SERS and EIS measurements by incorporating ZnO nanorod growth with electroplating

**DOI:** 10.1038/srep16454

**Published:** 2015-11-12

**Authors:** Xianli Zong, Rong Zhu, Xiaoliang Guo

**Affiliations:** 1 State Key Laboratory of Precision Measurement Technology and Instruments, Department of Precision Instrument, Tsinghua University, Beijing 100084, China

## Abstract

In this paper, a fine gold nanostructure synthesized on selective planar microelectrodes in micro-chip is realized by using an advanced hybrid fabrication approach incorporating growth of nanorods (NRs) with gold electroplating. By this developed nanostructure, integration of *in-situ* surface-enhanced Raman spectroscopy (SERS) detection with electrochemical impedance spectroscopy (EIS) measurement for label-free, nondestructive, real-time and rapid monitoring on a single cell has been achieved. Moreover, parameters of Au nanostructures such as size of nanoholes/nanogaps can be controllably adjusted in the fabrication. We have demonstrated a SERS enhancement factor of up to ~2.24 × 10^6^ and double-layer impedance decrease ratio of 90% ~ 95% at low frequency range below 200 kHz by using nanostructured microelectrodes. SERS detection and *in-situ* EIS measurement of a trapped single cell by using planar microelectrodes are realized to demonstrate the compatibility, multi-functions, high-sensitivity and simplicity of the micro-chip system. This dual function platform integrating SERS and EIS is of great significance in biological, biochemical and biomedical applications.

Surface-enhanced Raman spectroscopy (SERS) detection and electrochemical impedance spectroscopy (EIS) measurement have attracted great attention due to their promising advantages of rapid diagnosis, nondestructive and easy handling in analytical chemistry, characterization of trace chemical species, life science, and medical science^1^,^2^,^3^,^4^.

Raman spectrum as a “whole organism fingerprint” assessment can reveal detailed information about DNA, protein, lipid content and macromolecule conformations, which has been used for cancer detection, study of the molecular dynamics of cells undergoing mitoticdivision, mapping the distributions of cellular components and the immunoresponse investigation of granulocyte cells^5^,^6^,^7^,^8^. EIS is another powerful method for analyzing the electrical properties of a bio-system and is sensitive to surface phenomena and changes of bulk properties^9^. Cellular impedance measurements have been used in monitoring cell response to drug treatment, cell adhesion, cell death and cell cycle^10^,^11^,^12^,^13^. As real-time, label-free, *in-situ* and nondestructive tool, combination of SERS and EIS would enable us to comprehensively understand cellular activities and interaction between cell and environment.

To obtain highly sensitive SERS detection, numerous fabrication approaches have been developed to construct metal nanostructures, such as electrochemical synthesis^14^, metal colloid preparation^15^, nanoparticle self-assembly^16^, E-beam lithography^17^, vapor-liquid-solid (VLS) growth^18^ and etc. Although these methods have synthesized nanostructures on substrate with high scattering enhancement factor, most of them are not compatible with pre-fabricated micro-chip. So it is a great challenge to fabricate nanostructures selectively on target microelectrodes performing not only SERS detection but also *in-situ* impedance measurement. Microelectrodes used for single-cell analysis require small size. However, the double-layer impedance of the microelectrode existing in the electrode-electrolyte interface is inversely proportional to the electrode surface area. Downsizing of the electrode will unavoidably increase its double-layer impedance and thus degrade the sensitivity of the impedance measurement^19^. Fabricating nanostructures on microelectrodes can enlarge the effective surface area and therefore enhance the sensitivity of the EIS measurement.

It is well known that Au nanostructure is a good choice for both SERS and EIS due to its biocompatibility, excellent conductivity and enhancement on Raman scattering. Electrochemical synthesis of Au material (for example electroplating) on microelectrode can be conducted with good selectivity at room temperature. However, the solely plating process on the electrode cannot fabricate fine nanostructure (Fig. S1). In this paper, we propose a hybrid fabrication approach by incorporating growth of nanorods (NRs) with gold electroplating process, which can selectively construct fine gold nanostructure on target microelectrode for SERS detection and EIS measurement. Prior to electroplating, ZnO Nanorods are grown on the electrode via electro-hydrothermal process and served as a template of Au nanostructure. Compared with other template materials such as silicon or PS_18000_-b-PEO_7500_ micelle^20^,^21^, Zinc oxide (ZnO) nanorods are synthesized because they can be controllably grown on selected electrodes by using an electric-field assisted hydrothermal method at a gentle temperature which is compatible with pre-fabricated micro-chip^22^. Furthermore, ZnO forms a water-soluble complex with ammonium ion in the plating solution. The dissolution of ZnO NRs and the formation of Au nanostructure occur at the same time, and thus ZnO template can be completely removed in the process of electroplating without further treatment.

We previously developed a microchip with planar quadrupole-electrodes used for single-cell trapping based on negative dielectrophoresis and a pair of planar measuring electrodes at the center of quadrupole-electrode used for single-cell impedance measurement^23^.

In this paper, we synthesize Au nanostructure selectively on the center measuring electrodes (7 × 7 μm^2^) of the microchip by using the proposed hybrid fabrication approach to realize dual detections of *in-situ* SERS detection and EIS measurement for label-free, nondestructive, real-time and rapid monitoring on a single cell. Moreover, the SERS enhancement factor of up to ~2.24 × 10^6^ and double-layer impedance decrease ratio of 90% ~ 95% at low frequency range below 200 kHz have been achieved. The configuration of the micro-chip is shown in Fig. 1a. The synthesis process of Au nanostructure is shown in Fig. 1b,c, including growth of ZnO nanorods and formation of Au nanostructure via electroplating. Dual-modal *in-situ* measurements of SERS and EIS on single cell by using planar microelectrodes are realized as shown in Fig. 1d, which illustrate the versatility of the integrated micro-chip platform, rendering the technique valuable in both analytical chemistry and cell biology.

## Results and Discussion

### Characterization of ZnO Nanorods

The growth control of ZnO NRs selectively on target microelectrodes can be realized by applying DC or AC voltages onto the electrodes^22^. The position and direction of NRs are controlled by the local electric fields near the electrodes and the growth of ZnO NRs is promoted on the cathode due to participation of electrons. When a DC electric field is applied, ZnO NRs grow vertically on the cathode while the anode is stainless. When an electric field combining DC and AC (the peak-to-peak value of AC voltage is less than the magnitude value of DC voltage) is applied, ZnO NRs grow on the cathode with disorder direction while the anode is stainless also.

**Figure 1:**
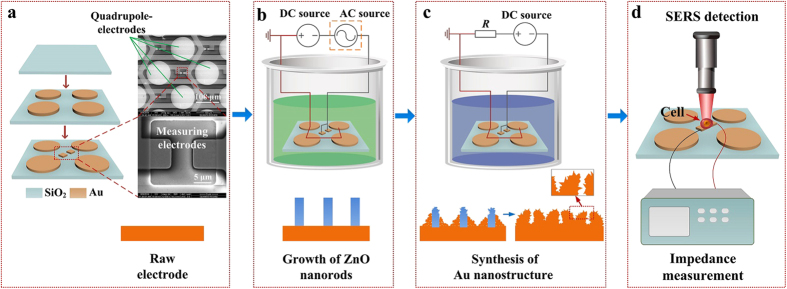
Configuration of the micro-chip, fabrication process of Au nanostructure and the dual-modal function of SERS and EIS measurements. (**a**) Configuration of micro-chip and SEM image of the prototype. Bottom SEM image is the higher resolution images of the dotted box showing the raw measuring electrodes. (**b,c**) Fabrication processes of Au nanostructure. (**d**) Dual-modal measurements of SERS and EIS for a single cell.

**Figure 2:**
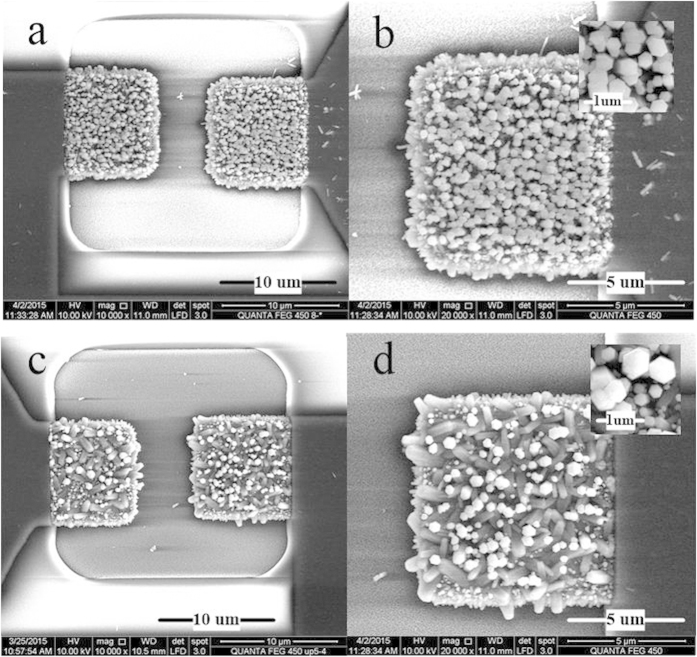
SEM images of ZnO NRs grown on micro electrode surface. (**a**) ZnO NRs grown under DC electric field. (**c**) ZnO NRs grown under DC-AC electric field. (**b**,**d**) are higher resolution SEM images of (**a**,**c**), respectively.

The micro-chip shown in Fig. 1a is adopted. Using the quadrupole-electrode as the anode and the center microelectrode pair as the cathode shown in Fig. 1b, ZnO NRs grow only on the measuring electrodes with different morphologies under DC control and DC-AC control respectively. Figure 2 shows scanning electron microscopy (SEM) images of ZnO NRs grown by DC (Fig. 2a,b) and DC-AC (Fig. 2c,d) control methods. By using DC control, ZnO NRs with average length of 0.5 μm and average diameter of 300 nm (listed in Table 1) grow vertically on the measuring electrode. While using DC-AC control, ZnO NRs with average length of 0.9 μm and average diameter of 500 nm grow disorderly on the measuring electrode. The EDX spectrum of the NRs (Fig. S2) indicates the material of ZnO.

### Characterization of Au Nanostructures

**Figure 3:**
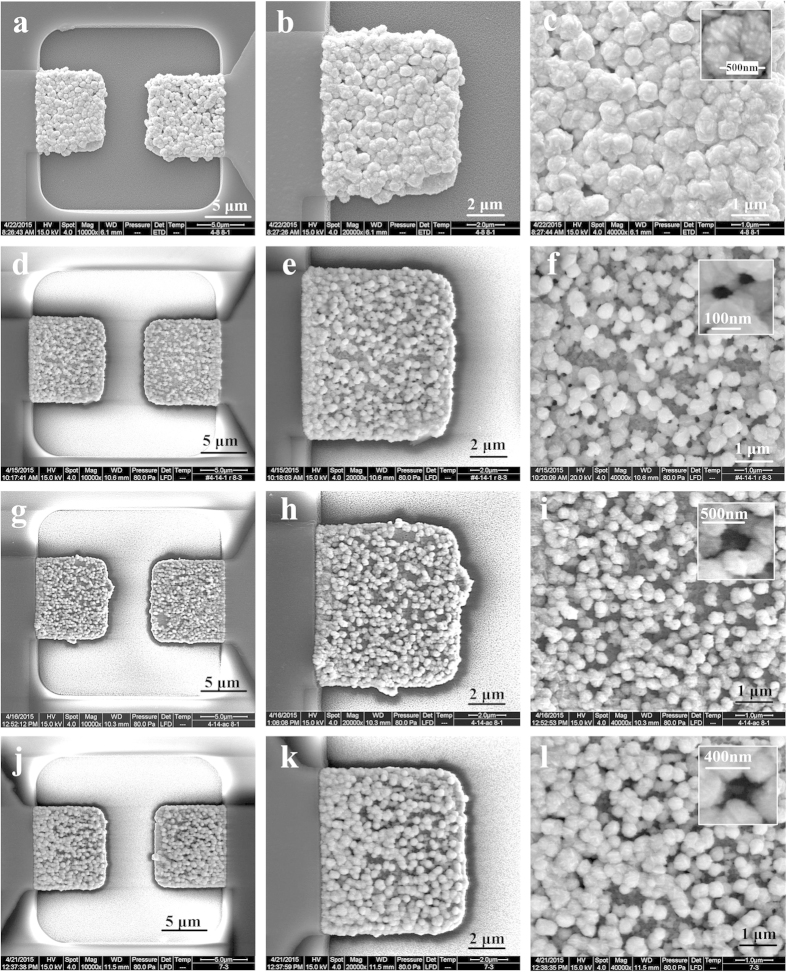
SEM images of Au nanostructures (Structure 1, 2, 3, 4) grown on the measuring electrode. (**a–c**) Structure 1 was constructed by electroplating for 130 s with NRs grown by DC control. (**d–f**) Structure 2 was constructed by electroplating for 70 s with NRs grown by DC control. (**g–i**) Structure 3 was constructed by electroplating for 60 s with NRs grown by DC-AC control. (**j–l**) Structure 4 was constructed by electroplating for 70 s with NRs grown by DC-AC control.

In gold electroplating process, a certain negative voltage (−0.87 V) is applied onto the measuring electrodes over a period of time while the quadrupole-electrode is grounded, as shown in Fig. 1c. The formation of Au nanostructures on the measuring electrodes is contemporaneous with dissolution of ZnO nanorods. Au nanostructures with different morphologies are constructed by adjusting the reaction time of electroplating and the growth of ZnO NRs. Figure 3 shows SEM images of different Au structures with different morphologies. Structure 1 with a morphology of nano-hillock structure (Fig. 3a–c) is constructed by using a plating time of 130 s onto the electrodes with ZnO NRs grown by DC control. Structure 2 with a morphology of nano-porous structure (Fig. 3d–f) is constructed by using a plating time of 70 s onto the electrodes with ZnO NRs grown by DC control. The inset image in Fig. 3f shows the nanohole of the porous structure with a diameter of ~50 nm formed by the dissolution of ZnO NRs (Fig. 2a,b). Structure 3 with a morphology of nano-forest (Fig. 3g–i) is constructed by using a plating time of 60 s onto the electrodes with ZnO NRs grown by DC-AC control. The inset image in Fig. 3i shows the gap of the nanoforest with a distance of ~400 nm formed by the dissolution of ZnO NRs (Fig. 2c,d). Structure 4 with a morphology of nano-forest (Fig. 3j–l) is constructed by using a plating time of 70 s onto the electrodes with ZnO NRs grown by DC-AC control. The inset image in Fig. 3l shows the gap of the nanoforest with a distance of ~320 nm formed by the dissolution of ZnO NRs (Fig. 2c,d). The EDX spectrum of the nanostructures (Fig. S3) demonstrates a fine Au material and indicates that ZnO NRs are dissolved completely after electroplating process.

The characteristics of constructed Au nanostructures are summarized in Table 2. With the increase of electroplating time from 60 s to 70 s and further to 130 s, the average size of Au nanoparticles increases from ~250 nm (Structure 3, Fig. 3i), to ~380 nm (Structure 2, Fig. 3f and Structure 4, Fig 3l) and further to ~550 nm (Structure 1, Fig. 3c). For Structure 1 and Structure 2, with the increase of electroplating time from 70 s to 130 s, the average size of nanoholes/nanogaps decreases from ~50 nm to ~30 nm. As for Structure 3 and Structure 4, with the increase of electroplating time from 60 s to 70 s, the average size of nanoholes decreases from ~400 nm to ~320 nm. These results shows that increasing plating time can increase the size of nanoparticles and decrease the size of nanoholes/nanogaps.

The nanoscale spaces, crevices or holes in or between nanoparticles contribute to the formation of hot spots which enhances SERS sensitivity due to near-field enhancement effects^1^,^24^. And nanostructures in sub-100 nm range and the nonspherical shapes have been reported to be ideal structures for SERS performances^2^,^25^. Therefore, Structure 1 with nanogaps of ~30 nm and Structure 2 with nanoholes of ~50 nm are expected to be ideal for SERS detection. In addition, the edges and corners on the rugged surface of the constructed nanostructures form a lot of hot spots which will enhance the SERS sensitivity greatly. Structure 3 has the smallest particle size due to the shortest electroplating time. With same Au plating time, Structure 2 and Structure 4 have approximately same particle size, but the nanoholes of Structure 4 reaches 320 nm, which will degrade SERS performance. Based on the above analysis, Structure 1, 2 and 3 are selected to conduct the following SERS and EIS experiments.

### SERS Properties of Au Nanostructures

**Figure 4:**
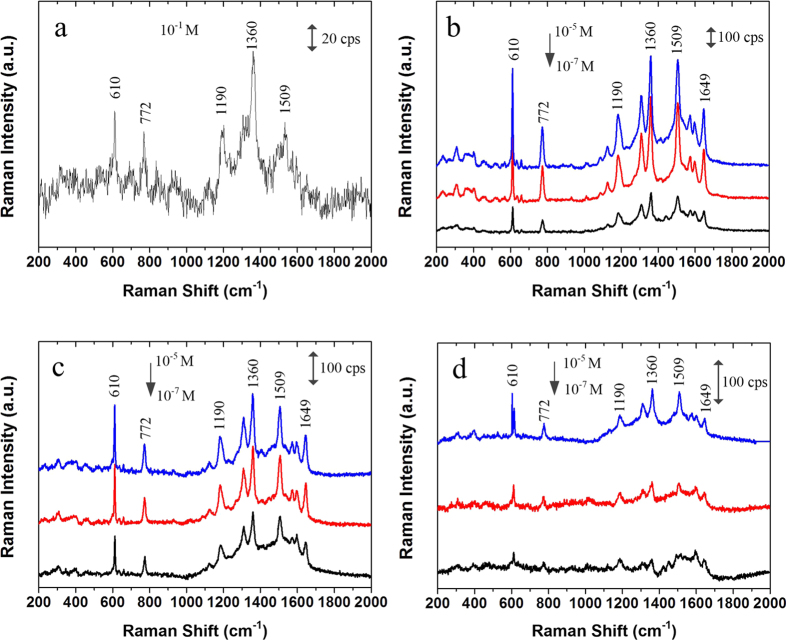
Raman spectrums of R6G solution. (**a**) Normal Raman spectrum of 10^−1^ M R6G solution using the raw electrodes without nanostructure. SERS spectrums of R6G (10^−5^ M to 10^−7^ M) using (**b**) Structure 1, (**c**) Structure 2, and (**d**) Structure 3. Excitation wavelength: 633 nm; laser power: 3 mW; integrating time: 15 s.

SERS performances of Au nanostructures are demonstrated through a series of experiments conducted by using R6G as a target molecule. Figure 4 shows the Raman spectrums of R6G solution by using and without using Au nanostructured microelectrodes. Figure 4a shows the normal Raman spectrum of R6G solution with 10^−1^ M adsorbed on the raw measuring electrode surface without Au nanostructures (Fig. 1a). Figure 4b–d are SERS spectrums of R6G (10^−5^ M to 10^−7^ M) adsorbed on the nanostructured electrodes with Structure 1 (Fig. 4b), Structure 2 (Fig. 4c), and Structure 3 (Fig. 4d), respectively. With the increase of R6G concentration, the intensity of SERS signal is increased. The Raman bands at about 610, 772, 1190, 1360, 1509 and 1649 cm^−1^ are attributed to R6G which agree well with other reported data^26^.

In order to investigate the SERS effects of Au nanostructured electrodes, the enhancement factors (EFs) are calculated using the following equation^27^


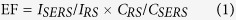


where 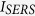 and 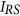 are the Raman intensities of R6G molecules adsorbed on the electrode with and without Au nanostructure. 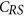 is the concentration of R6G solution for normal Raman spectrum measurement (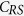 = 10^−1^ M) and 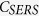 is the concentration of R6G solution for SERS measurement (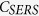 = 10^−7^ M).

The EFs of three Au nanostructured electrodes are calculated as 2.5 × 10^6^ (Structure 1), 2.24 × 10^6^ (Structure 2) and 0.86 × 10^6^ (Structure 3), respectively. Structure 1 has the highest EF, Structure 2 is second and Structure 3 has the lowest EF. EFs of Strucure 1 and 2 are larger than other nanostructured samples previously reported^21^,^26^. As summarized in Table 2, the average gap/hole sizes of Structure 1 and Structure 2 are ~30 nm and ~50 nm respectively, these nanoscale holes and gaps provide a large number of hot spots. As for Au nanostructure 3, the particle size and gap size all reach hundreds of nanometers, which limits the formation of hot spots and thus degrades SERS effect.

### Impedance Measurement of Au Nanostructures

**Figure 5:**
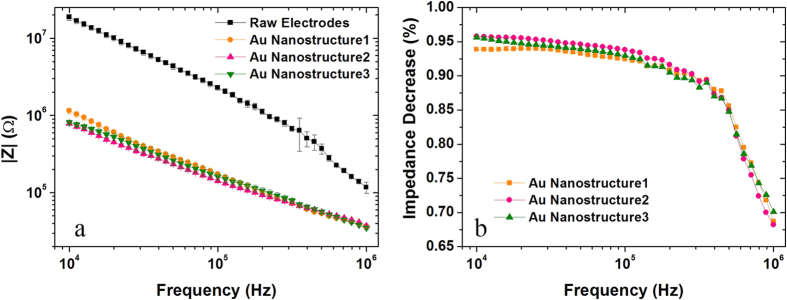
Impedance measurement of NaCl solution. (**a**) Impedance measurement results using the measuring electrodes with and without nanostructures in 0.9% NaCl solution. (**b**) Impedance decrease ratios of three nanostructured electrodes compared with the raw electrode without nanostructure.

Decreasing double-layer impedance in the electrode-electrolyte interfaces can enhance the sensitivity of impedance measurement on a single cell by using microelectrodes. Therefore, impedance measurement using the measuring electrode pair with three Au nanostructures were conducted, respectively. The double-layer impedances in the electrode-electrolyte interfaces of the electrodes with and without Au nanostructures are experimentally tested in 0.9% NaCl solution. As shown in Fig. 5, the tested impedances using the nanostructured electrodes decrease dramatically compared with the raw electrode without nanostructure. Impedance decrease ratio achieves 90% ~ 95% at low frequency range below 200 kHz. The impedance decrease is attributed to the reduction of the double-layer impedance, induced by effective surface area enhancement of the electrodes in virtue of Au nanostructure. Among the three Au nanostructures, structure 2 has the highest impedance decrease ratio while structure 1 has the lowest decrease ratio.

The criterion to recognize an ideal nanostructure for SERS detection and *in-situ* EIS measurement is that the structure possesses the largest Raman EF and the lowest double-layer impedance. The Raman EF and impedance decrease ratio (indicates double-layer impedance decrease) at 100 kHz of Structure 1, 2, and 3 were summarized in Table 2. Among the three Au nanostructures, Structure 1 has the highest Raman EF (2.5 × 10^6^) while its impedance decrease is the lowest (96%). The EF of Structure 2 is slightly lower (2.24 × 10^6^) while its impedance decrease ratio is the highest (98%). Considering enhancement efficiency of both SERS and impedance measurements, Structure 2 is finally selected as the ideal structure to conduct the dual-modal detection of a single Hela cell.

### Dual-modal Detection of Single Hela Cell

Noninvasive detection of cellular Raman spectrum and *in-situ* impedance measurement onto single cell by using planar microelectrodes are greatly challenging. Planar microelectrodes can provide an ideally noninvasive approach to detect cells and may allow high throughput by means of scaled up array structure. However, using planar microelectrodes to detect single-cell impedance has rarely been demonstrated because of the problem of the large double-layer impedance. In this paper, we solve this problem and demonstrate dual detections of SERS and EIS on single-cell using our micro-chip with nanostructured planar measuring electrodes.

Another challenge in dual-modal detection is how to position cells onto the target measuring electrodes. Cell manipulation and position have been realized using negative dielectrophoresis (nDEP) forces generated by applying electric signals onto the quadrupole-electrodes, the details of which can be found in our previous paper^23^. Before SERS detection and EIS measurement, living cells were trapped onto the measuring electrodes by nDEP forces generated from quadrupole-electrodes.

According to recent reports, the optimal excitation wavelength of 700–1100 nm is preferred in Raman detection of biological tissue for little light scattering, absorption, and fluorescence^28^,^29^. In this paper, Raman signals comes from the interface between cell and Au nanostructures on electrode surfaces, which means light scattering and absorption have very little impact on the Raman detection of cell. Moreover, Hela cells are cultured without fluorescent marker to avoid the interference of fluorescence. Based on the above considerations, excitation wavelength of 633 nm is adopted in the experiment by reasonable control over the laser power and integrating time to obtain better signals.

**Figure 6:**
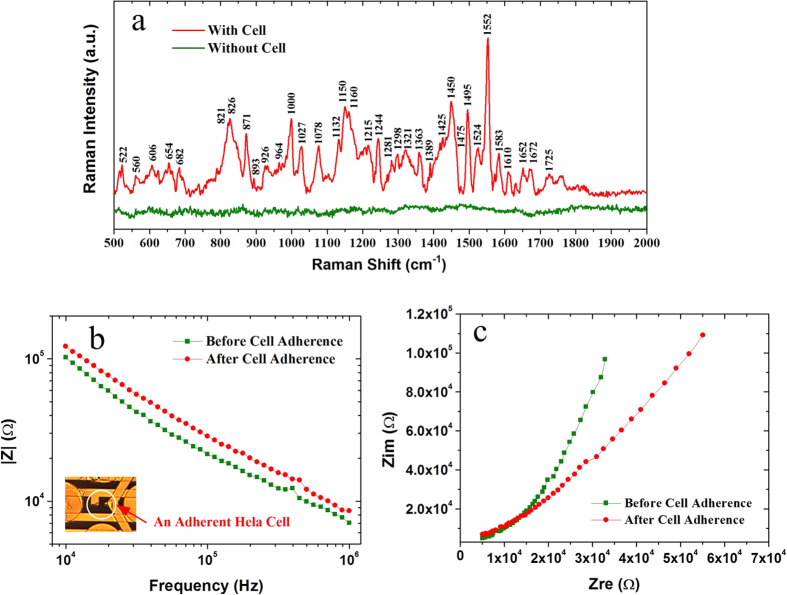
Dual-modal detection of a single Hela cell. (**a**) SERS spectrum of a single Hela cell adhering on the underneath electrode with Au nanostructure (Structure 2). (**b**) Comparison of the cellular impedances before and after its adherence. Inset in (**b**) is the light microscope image of a single Hela cell adhering on the measuring electrodes. (**c**) Cole-Cole plots of the impedance measurements.

Figure 6a shows the SERS spectrum obtained from a single Hela cell adhered on the measuring electrodes with Au nanostructure 2. The cellular Raman spectrum is greatly enhanced by the nanostructure on the electrode underneath the cell. Compared with the Raman spectrum of the background without cell, the cellular Raman peak assignments are analyzed thoroughly and Table S1 demonstrates the existence of lipid, protein and carbohydrate on cell membrane^5^,^30^,^31^. Besides SERS detection, an *in-situ* impedance measurement is conducted by using the measuring electrodes. The impedance spectrums of single living Hela cell before and after its adherence are shown in Fig. 6b,c. It is seen that the magnitude of the celluar impedance increases by about 28% from its suspension to adherence, which agrees with the fact that the space volume between the cell and the underneath electrodes is compressed when the cell is adhering onto the electrodes.

SERS detection presented in this paper can reveal the information at the interface between cell and Au nanostructure on the electrodes. However, the enhancement range cannot reach cells’ nucleus. Moreover, SERS and EIS detections reveal the whole message of a single cell and the mapping of a single cell cannot be obtained. Nevertheless, the proposed approach provides *in-situ*, long-time and nondestructive SERS detection and EIS measurement of a single cell simultaneously, which can be used as a complementary tool compared with intracellular SERS detection.

Nowadays, multi-mode measurement is an inevitable trend in study of cells and biological tissues^32^,^33^. With the development of SERS technologies, easy-handling, low cost and controllability and reproducibility are important requirements for substrate production^34^,^35^. The gold plating technique incorporated with ZnO NRs growth can fabricate fine Au nanostructure on microelectrodes with high controllability and low-cost. Moreover, fabricating nanostructures selectively on the target microelectrodes is critical for the microchip with multiple functions. In addition, the integration of SERS detection and EIS measurement provides us not only Raman spectra, but also the electrical parameters of a single cells. In general, this dual function platform integrating SERS and EIS is of great significance in biological, biochemical and biomedical applications.

## Conclusions

In this paper, a fine Au nanostructure selectively synthesized on planar microelectrodes in micro-chip is realized by using an advanced hybrid fabrication approach incorporating growth of nanorods (NRs) with gold electroplating. Au nanostructured electrodes allows integrating SERS detection with *in-situ* EIS measurement for label-free, nondestructive, real-time and rapid monitoring on molecules and cells. For the first time, SERS detection and *in-situ* EIS measurement on single Hela cell by using the planar microelectrodes are realized to validate dual functions of the micro-chip. The proposed method also allows high through-put analyses on cells and molecules with real-time, *in-situ* SERS and EIS detection by means of scaled up array structure in a micro-chip.

## Methods

Chemical reagents were purchased from Aladdin. Morphologies of the samples were characterized by using SEM (CSM-950) with energy-dispersive x-ray spectroscopy (EDX). The cell image was taken by using light microscope (DM2500, Leica, Germany) with a CCD (DFC450C, Leica, Germany).

### Fabrication of Micro-chip

The detailed fabrication of the micro-chip can be found in our previous publication^23^. The micromachining process of the micro-chip is shown in Fig. 1a. Wire bonding was used to connect the electrodes with outside pins.

### Synthesis of ZnO Nanorods

ZnO NRs locally and selectively grown on measuring electrodes was synthesized using wet chemical method with electric-field assistance as shown in Fig. 1b. The measuring electrodes serve as the cathode while the quadrupole-electrodes serve as the anode. The whole sample was immerged in equal molar aqueous solution (0.015 M) of Zn(NO_3_)_2_·6H_2_O and HMTA (C_6_H_12_N_4_) heated at 75 °C. In DC control of ZnO NRs growth, the measuring electrodes were connected with a negative DC voltage of −0.4 V (generated by DC power supply DH1715 A-3) and the quadrupole-electrodes were connected with GND. In AC-DC control, the measuring electrodes were connected with an AC sine-wave voltage (1 MHz, 0.2 Vpp, generated by Tektronix Arbitrary/Function Generator AFG 3252) with an offset of −0.4 V. The quadrupole-electrodes were connected with GND. After 1h, the micro-chip was picked up from the solution, rinsed with deionized water using ultrasonic cleaning and dried in air.

### Process of Gold Electroplating

As shown in Fig. 1c, gold electroplating process was further conducted to realize the synthesis of Au nanostructures on the measuring electrodes at room temperature. The gold plating solution contains gold trichloride (AuCl_3_, 0.08 ~ 0.11 M), sodium sulfite heptahydrate (Na_2_SO_3_∙7H_2_O, 0.4 ~ 1.0 M), ammonium citrate tribasic ((NH_4_)_3_C_6_H_5_O_7_, 0.28 ~ 0.37 M) and EDTA (0.17 ~ 0.31 M). During the electroplating process, negative voltage of −0.87 V was applied to the measuring electrodes and the quadrupole-electrodes were grounded. Plating voltages of −0.86 V and −0.88 V were also investigated and the results (not shown in this paper) demonstrated that a low magnitude of plating voltage would form flat surfaces on the electrodes while a high magnitude of voltage would decrease the stiffness of nanostructure. Therefore, −0.87 V was finally selected as the plating voltage. Four kinds of Au nanostructures were obtained by using different electroplating time and ZnO NRs.

### SERS Spectra Measurement of R6G

Raman measurements were conducted by using a confocal microscopy Raman Spectrometer (LabRam HR800) equipped with a 633 nm laser and a 100× objective to focus the laser light onto the sample surface and collect the scattered light. 20 μL of R6G solution with concentration varying from 10^−7^ M to 10^−5^ M was dripped onto the surface of three micro-chips with different Au nanostructures, respectively. SERS spectra were acquired immediately after the substrate was dried under ambient conditions. 20 μL R6G solution of 10^−1^ M was dripped onto the raw measuring electrode without nanostructure. A normal Raman spectrum of 10^−1^ M R6G solution was also acquired after the substrate was dried under ambient conditions. The laser power at the sample position was 3 mW, the laser beam size was about 10 μm, and the integrating time was 15 s with once accumulation.

### Impedance Measurement of NaCl

30 μL NaCl solution with concentration of 0.9% was dripped on the surfaces of three micro-chips with three different nanostructures, respectively. The real-time impedance measurements were conducted by using the measuring electrode pairs connecting with an electrochemical workstation (PARSTAT4000, Princeton Applied Research, USA).

### Cell Seeding and Detection

Human carcinoma (HeLa) cells were cultured as a monolayer in a 60 mm Petri dish containing Dulbecco’s Modified Eagle Medium (DMEM) supplemented with 10% fetal bovine serum at 37 °C and 5% CO_2_ atmosphere. The cells were harvested from the Petri dish at the log phase of growth by 0.25% trypsin/EDTA, and then were re-suspended in the culture medium at a concentration of 2 × 10^5^ cells mL^−1^. The suspended HeLa cells were poured into the sample pool of the micro-chip. The cells were manipulated and positioned at the site of the measuring electrodes using nDEP forces generated by applying electric signals onto the quadrupole-electrodes^23^. By using an appropriate concentration of cells (2 × 10^5^ cells mL^−1^), single cells could be trapped onto the measureming electrodes, the detailed process of which can be found in our previous paper^23^. The real-time impedance measurement was performed by using the measuring electrodes connecting with an electrochemical workstation during the single Hela cell adhereing onto the underneath electrodes and the Raman spectra was detected after the adherence of the cell. In Raman detection, the excitation wavelength was 633 nm, the laser power at the sample position was 3 mW, and the integrating time was 90 s with once accumulation.

## Additional Information

**How to cite this article**: Zong, X. *et al.* Nanostructured gold microelectrodes for SERS and EIS measurements by incorporating ZnO nanorod growth with electroplating. *Sci. Rep.* **5**, 16454; doi: 10.1038/srep16454 (2015).

## Supplementary Material

Supplementary Information

## Figures and Tables

**Table 1:** Growth condition and characteristics of ZnO NRs.

	Growthcontrol	DCvoltage[V]	ACvoltage[Vpp]	Growthtime[hour]	Averagelength[μm]	Averagediameter[nm]	Directionof ZnONRs
ZnO NRs 1	DC	−0.4	—	1	0.5	300	Vertical
ZnO NRs 2	DC-AC	−0.4	0.2	1	0.9	500	Disordered

**Table 2:** Fabrication condition and characteristics of Au nanostructures.

	ZnO growth control	Platingvoltage[V]	Platingtime[s]	Averageparticle size[nm]	Averagehole/gap size[nm]	EF	Impedance decreaseratio at100 kHz
Structure 1	DC	−0.87/GND	130	550	30	2.5 × 10^6^	96%
Structure 2	DC	−0.87/GND	70	380	50	2.24 × 10^6^	98%
Structure 3	DC-AC	−0.87/GND	60	250	400	0.86 × 10^6^	97%
Structure 4	DC-AC	−0.87/GND	70	380	320	—	—
