# Supplementary material for: Nanostructured gold microelectrodes for SERS and EIS measurements by incorporating ZnO nanorod growth with electroplating
Source: Sci Rep. 2015 Nov 12;5:16454. doi: 10.1038/srep16454 (PMC4642340; doi:10.1038/srep16454)
Supplement: Supplementary Information [file srep16454-s1.doc]

**Supplementary information**

**Title:** Nanostructured gold microelectrodes for SERS and EIS measurements by incorporating ZnO nanorod growth with electroplating

**Authors**: Xianli Zong, Rong Zhu* and Xiaoliang Guo

State Key Laboratory of Precision Measurement Technology and Instruments, Department of Precision Instrument, Tsinghua University, Beijing 100084, China


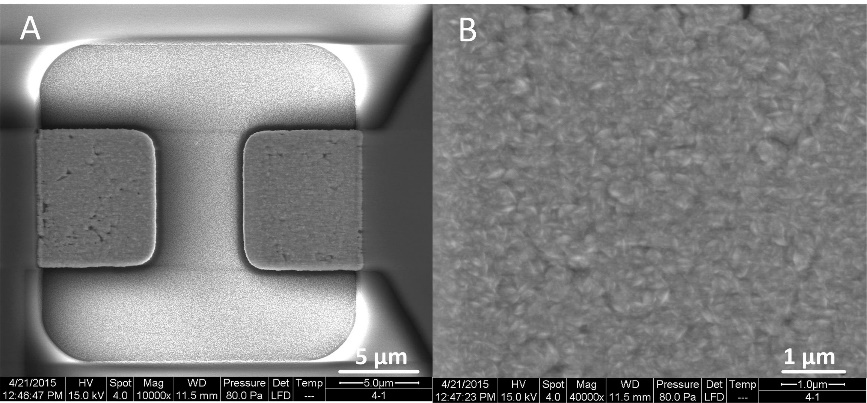


**Figure S1** SEM images of measuring electrodes surface after gold electroplating process without ZnO NRs as template. B) Enlarged SEM image of A).

**Figure S2.** EDX result of ZnO NRs.

**Figure S3** EDX results of Au nanostructure. The tested Si and O elements are from the micro-chip SiO_2_ insulation layer.


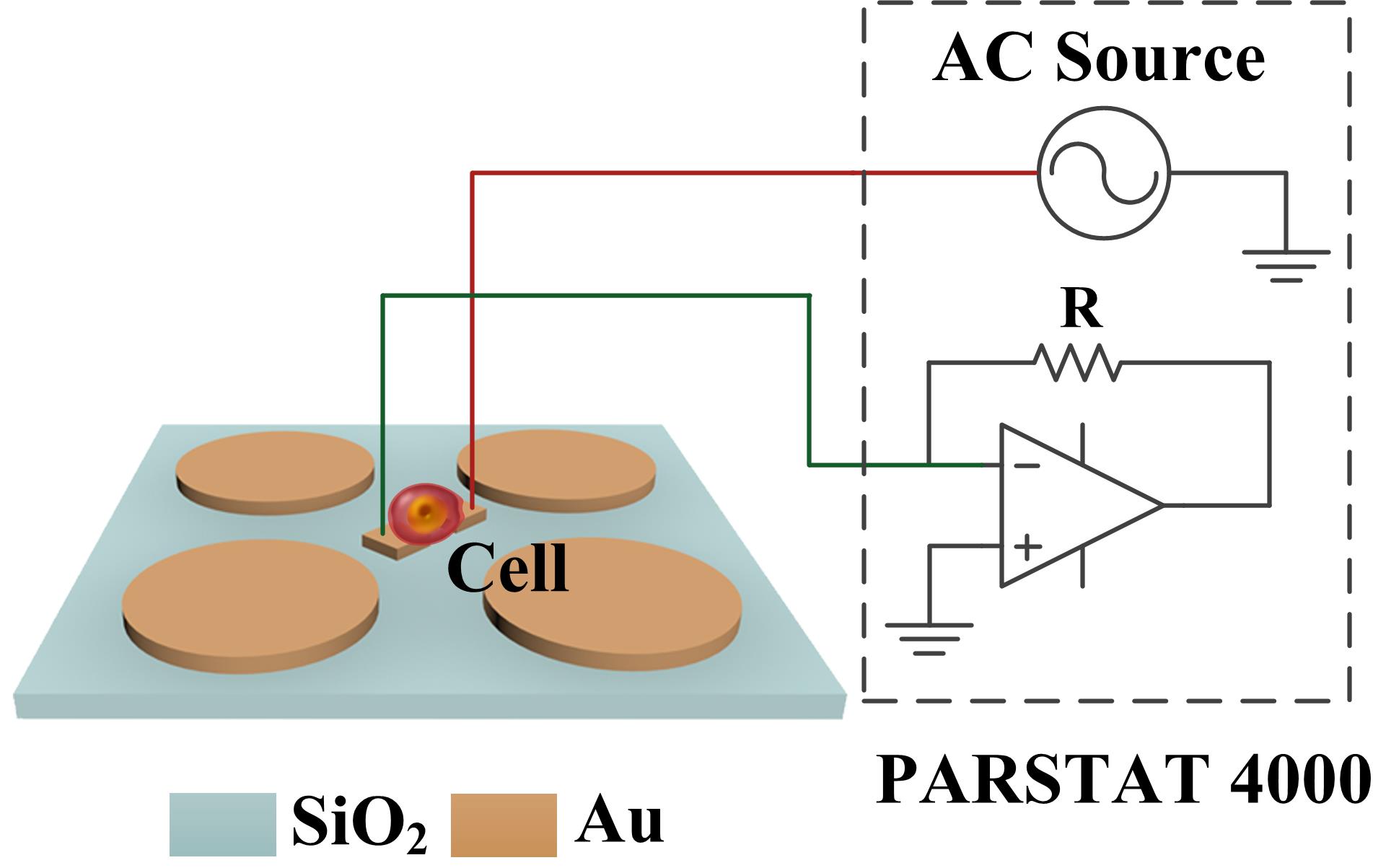


**Figure S4** Schema of cell impedance measurement. A single cell was positioned on the surface of the nanostructured measuring electrodes pair and the impedance signal was measured by using PARSTAT 4000.

Table S1 Raman frequencies and their assignments

| **Raman frequency in wavenumber/cm^-1^** | **Assignment** | | |
| --- | --- | --- | --- |
|  | **Protein** | **Carbohydrate** | **Lipid** |
| 515 | S-S str |  |  |
| 522 | S-S str |  |  |
| 538 | S-S str |  |  |
| 560 |  | GluA, Glc |  |
| 606 |  | Man |  |
| 623 | Phe |  |  |
| 646 | Phe |  |  |
| 654 | C-S str |  |  |
| 662.5 | C-S str |  |  |
| 682 |  |  |  |
| 821 | Tyr |  |  |
| 826 |  | Man |  |
| 830 | Tyr |  |  |
| 848 | Tyr |  |  |
| 871 | Trp |  |  |
| 893 | C-C str. |  |  |
| 926 | C-C str. | Man |  |
| 964 |  |  |  |
| 1001 | Phe |  |  |
| 1027 | Phe |  |  |
| 1078 | C-N | Glc, GluA, Man | C-O C-O-C str |
| 1094 | O-P-O sym. str. |  | C-N str |
| 1132 |  |  | C-N str |
| 1150 | Amide II |  |  |
| 1160 | Amide I |  |  |
| 1208 | Amide III |  |  |
| 1215 | Amide III |  |  |
| 1244 | Amide III |  |  |
| 1270 | Amide III |  |  |
| 1281 | Amide III |  |  |
| 1298 | Amide III |  |  |
| 1321 |  | GlcNac |  |
| 1363 | Trp |  |  |
| 1389 |  |  |  |
| 1418 |  |  | C-H |
| 1425 |  |  | C-H |
| 1432 | Trp | GlcNac |  |
| 1450 | C-H_2_ bend | C-H_2_ bend |  |
| 1475 |  |  |  |
| 1495 |  |  |  |
| 1524 |  |  |  |
| 1539 |  |  |  |
| 1552 | Amide II, Trp | GlcNac |  |
| 1572 |  |  |  |
| 1583 | Trp |  |  |
| 1610 | Tyr, Phe |  |  |
| 1631 |  | GlcNac |  |
| 1652 | Amide I |  |  |
| 1672 | C=O str |  |  |
| 1725 | C=O str |  |  |

Abbreviations: str, stretching; sym, symmetric; Tyr, tyrosine; Trp, tryptophan; Phe, phenylalanine; Man, D-mannose; Glc, D-glucose; GluA, glucuronic acid and GlcNac, N-acetyl-D-glucosamine.

Assignments are based on studies ^5, 30, 31^ in References.
